# Annotations

**Published:** 1904-10-01

**Authors:** 


### Annotations.

### Nervine Stimulants and Human Progress.

The columns of the daily press during the last few days have exhibited certain figures which are not without significance in relation to some tendencies of modern life. These are to be found in the Excise returns and show a very considerable decrease of the amount of wine consumed during the past year and with this a marked increase in the consumption of tea. Less wine would appear to have been drunk in 1903 than in any single year during the previous decade and compared with 1902 the figures show a fall of no less than 12 per cent. Beer and spirits have also continuously declined since 1899, which was their record year. On the other hand, the use of tea continues to make steady progress. The figures show a growing increase since 1890 so that in 1903 the average consumption per head of the population was over six pounds, which is nearly a pound per head more than was used in 1890. When the figures relating to alcohol are contrasted with those regarding tea, it appears manifest that there is an increasing recognition of the view that modern conditions make the use of the former less and less necessary. But, in one form or other, civilised human beings demand agents which exercise a stimulating influence on the nervous system, and the duty of the physiologist is to explain this habit rather than to condemn it. According to a recent lecture by Mr. Jonathan Hutchinson, such agents, not excluding alcohol, have played a large part in the development of the human brain, and it has only been on the discovery of one or other of them that any nation has shown much aptitude for progress. Tea and coffee are aids to mental effort, and the question whether they should or should not be classed as foods may be left to the makers of definitions and to those whose doctrines suggest that wise feeding and drinking have only become possible since chemical physiology has made its latest calculations.

### Marriage and Tuberculosis.

Always interesting, the marriage question is very prominent at the present moment. We have no idea of entering into the controversy which has been excited by the extraordinary prediction of Mr. George Meredith that marriage will one day be allowed by the State for a certain period. If he were not an eminent novelist, the suggestion would not be worth notice, and Canon Duckworth is probably correct in his assumption that Mr. Meredith has either not given the matter much consideration or does not intend his prophecy to be taken seriously. Very seriously indeed, however, we protest against certain unions which are quite permissible by law, and upon which the Church pronounces her benediction. The particulars of the tragic suicide at one of the great London hotels, last week, supply a singularly painful and pathetic argument against the marriage of persons afflicted with tuberculosis. The widow of the deceased, who was a native of America, stated in her evidence that when she married him on August 6th she was aware that he was suffering from that complaint. She had since learned that he was addicted to the use of morphia. How far it was due to his malady is not quite clear, but as his widow affirms that on several occasions he remarked that he would take his own life rather than die of a lingering disease, the connection between the two may be assumed. The probability is that he became a victim to the morphia habit in the hope that it would prevent him from dwelling upon the fate that seemed inevitable. But the point we desire to emphasise is that neither man nor woman affected with active tuberculosis should think of getting married. Such marriages can scarcely fail to have more or less disastrous results; if not an offence to the individual, they are a transgression of the law of health which, after all, is an integral portion of the law of God.

### The Japanese in Formosa.

The island of Formosa, so called by the earliest Spanish settlers as a description of its surpassing beauty, has for the last two or three hundred years, even if not for a longer period, appeared to justify the well-known words of the missionary hymn by affording at least one example of a place where only man was vile. During the greater part of the nineteenth century it was nominally under the rule of China, but was scarcely, if at all, a source of revenue to its supposed possessors. A few Chinese settlers inhabited the coast line, and the interior of a country half the size of Ireland was given over to bloodthirsty and cannibal savages, by whom all law and government were set at defiance. By the treaty of Shimonoseki, in 1895, the island was ceded to Japan as a portion of the spoils of victory; and the *Times* of last Saturday contained a highly interesting account of the complete transformation which has been effected by the victors, who, indeed, seem to have nothing to learn in the way either of colonisation or of the government of subject nations. The savage inhabitants of the interior have been completely subdued, so that life and property are secure in every part of the island. Schools have been established in every district, roads and railroads have been made and more are projected, telegraphs, telephones, post offices, and savings banks have been opened, and are eagerly and increasingly employed. The revenue has increased tenfold in nine years, and one of the most fertile countries in the world is rapidly being brought under complete cultivation. But perhaps the most noteworthy portion of this transformation is that which has relation to public health. Not only are sanitation and water-supply enforced by the Japanese, but hospitals have been established in nine towns which are mentioned by name in the *Times*, as well as "branch hospitals in less important places." A medical college has also been founded and set to work, and is resorted to both by native and by Chinese students. More wonderful still, the Government has boldly grappled with the native vice of opium-smoking, which is strictly prohibited among the Japanese themselves. The supply of opium has been made a Government monopoly, and the drug is only sold to registered and licensed persons, who are thus formed into a class analogous to habitual drunkards among ourselves, but with the difference that they are much better and more effectively controlled. No new licenses will be permitted to issue, and the number of licenses has diminished by 17,000 in 18 months. There can be no doubt that the practice will soon be extinguished; and the Japanese Government does not hesitate to sacrifice a considerable revenue in order to preserve and improve the health of its subjects.

